# Reviewer acknowledgement 2015

**DOI:** 10.1186/s12931-016-0331-x

**Published:** 2016-02-04

**Authors:** Jan Lötvall, Reynold A. Panettieri

**Affiliations:** Göteborg University, Göteborg, Sweden; Rutgers, The State University of New Jersey, New Brunswick, USA

## Abstract

The editors of *Respiratory Research* would like to thank all of our reviewers who have contributed to the journal in Volume 16 (2015).

**Ian Adcock**

UK

**Yochai Adir**

Israel

**Saurabh Aggarwal**

USA

**Anurag Agrawal**

India

**Stefano Aliberti**

Italy

**Isaac Almendros**

Spain

**Kjell Alving**

Sweden

**Andre FS Amarala**

UK

**Yassine Amrani**

UK

**Steven An**

USA

**Stefan Andreas**

Germany

**Michael Arzt**

Germany

**Koichiro Asano**

Japan

**Elena Atochina-Vasserman**

USA

**Joan A Barbera**

Spain

**Kenneth K Barfod**

Denmark

**Peter Barnes**

UK

**Elizabeth Barry**

USA

**Elizabeth Bartolak-Suki**

USA

**Kai-Michael Beeh**

Germany

**Christoph Beisswenger**

Germany

**Kylie Belchamber**

UK

**Andjelo Beletic**

Serbia

**David bennett**

Italy

**John Bentley**

USA

**Pankaj Bhavsar**

UK

**Andrea Bianco**

Italy

**Kimberly Bigelow**

USA

**Anna Birokova**

USA

**Mark Birrell**

UK

**Thomas Bitter**

Germany

**Francesco Blasi**

Italy

**Jessica Bon**

USA

**Jamie Bonner**

USA

**Sebastien Bonnet**

Canada

**Philippe Bonniaud**

France

**Mieke Boon**

Belgium

**Keren Borensztajn**

France

**Pieter Borger**

Switzerland

**Piera Boschetto**

Italy

**Graham Bothamley**

UK

**Robert Boughman**

USA

**Arnaud Bourdin**

France

**Armin Braun**

Germany

**Emilio Bria**

Italy

**Despina Briana**

USA

**Vito Brusasco**

Italy

**Marie Bruyneel**

Belgium

**Charles Burger**

USA

**Janette Burgess**

Netherlands

**Luigino Calzetta**

Italy

**Vinicius de Farias Carvalho**

Brazil

**Gianluca Casoni**

Italy

**Flemming Cassee**

Netherlands

**Didier Cataldo**

Belgium

**Lorenzo Cecchi**

Italy

**Jamila Chakir**

Canada

**James Chalmers**

UK

**Nan-Shan Chang**

Taiwan

**Anuran Chatterjee**

USA

**Ping Chen**

China

**Marco Chilosi**

Italy

**Antonio Cittadini**

Italy

**Jocelyn Claude**

USA

**Debbie Clements**

UK

**Harold Collard**

USA

**Marco Contoli**

Italy

**Rob P Coppes**

Netherlands

**Lorenzo Corbetta**

Italy

**Estelle Cormet-Boyakaa**

USA

**Diego Cortinovis**

Italy

**Vincent Cottin**

France

**Elizabeth Cowley**

Canada

**Alain Cuna**

USA

**Pranjali Dalvi**

USA

**Monique Dehoux**

France

**Yvonne Dempsie**

UK

**Deepak Deshpande**

USA

**Laurence Dewachter**

Belgium

**Massimo Di Maio**

Italy

**Fabiano Di Marco**

Italy

**Joshua Diamond**

USA

**Dolores Divizio**

USA

**Claire Doerschuk**

USA

**Louise Donnelly**

UK

**Claudio F Donner**

Italy

**Peter Dorfmuller**

France

**Linda Ekerljung**

Sweden

**Charles Emala**

USA

**Kerry Empey**

USA

**Susanna Esposito**

Italy

**Rachael Evans**

UK

**Leo Fabbri**

Italy

**Aurelie Fabre**

Ireland

**Jeremy Falk**

USA

**Rosa Faner**

Spain

**Terence Flotte**

USA

**Stefano Fogli**

Italy

**Nicholas Robert Forsyth**

UK

**Matthew Foster**

USA

**Karl Franklin**

Sweden

**Luca Gallelli**

Italy

**Judith Garcia-Aymerich**

Spain

**Ignacio Garcia-Verdugo**

France

**Eric Garshick**

USA

**Gail Gauvreau**

Canada

**Moyar Qing Ge**

USA

**Joaquim Gea**

USA

**Francesco Gelsomino**

Italy

**Patrick Geraghty**

USA

**William Gerthoffer**

USA

**Adam Giangreco**

UK

**Alison Glasser**

USA

**Neil Goldenberg**

Canada

**Nira Goldstein**

USA

**Lynda Goodfellow**

USA

**Reinoud Gosens**

Netherlands

**Andrew Gow**

USA

**Lidwien Graat-Verboom**

Netherlands

**Catherine Greene**

Ireland

**Eduard Guasch**

Spain

**Andreas Guenther**

Germany

**Benoit Guery**

France

**Shicheng Guo**

China

**Nishant Gupta**

USA

**Robert Guzy**

USA

**Tillie Hackett**

Canada

**Mehra Haghi**

USA

**James Hagood**

USA

**John Haley**

USA

**Wayne Hall**

Australia

**Oskar Hallgren**

Sweden

**Alf Hamann**

Germany

**Jorg Hamann**

Netherlands

**Gerhard Hamilton**

Austria

**Masayuki Hanaoka**

Japan

**Shu Hashimoto**

Japan

**Louise Hecker**

USA

**Linnea Hedman**

Sweden

**Irene Heijink**

Netherlands

**John A Heit**

USA

**Marc Hershenson**

USA

**Steven Hill**

USA

**Sandy Hodge**

USA

**Anne Holland**

Australia

**Alex Horsley**

UK

**H Dean Hosgood**

USA

**Jian-An Huang**

USA

**Tomoko Ichiki**

USA

**Masakazu Ichinose**

Japan

**Hiromasa Inoue**

Japan

**Kazuhiro Ito**

UK

**Frode Jahnsen**

Norway

**Anna James**

Sweden

**Sabina Janciauskiene**

Germany

**Luke Janssen**

Canada

**Gisli Jenkins**

UK

**Jeffrey Jennings**

USA

**Chang Jiang Guo**

USA

**Jill Johnson**

UK

**Pavol Joppa**

Slovakia

**Stephane Jouneau**

France

**Anthony Jude**

USA

**Eleanor Kable**

Australia

**Ralf Kaiser**

Germany

**Noor Kalsheker**

UK

**Harry Karmouty-Quintana**

USA

**Christine Keenan**

Australia

**Barry Kennedy**

Ireland

**Brian Kent**

UK

**Farrah Kheramand**

USA

**Toshiaki Kikuchi**

Japan

**Gunseli Kilinc**

Turkey

**Victor Kim**

USA

**Dae Woo Kim**

South Korea

**Irah King**

Canada

**Loes Kistemaker**

Netherlands

**Alex KleinJan**

Netherlands

**Edward Knol**

USA

**Akira Koarai**

Japan

**Leo Koenderman**

Netherlands

**Martin Kohlhaufl**

Denmark

**Mirjam Kool**

Netherlands

**Jerry Krishnan**

USA

**Ramaswamy Krishnan**

USA

**Vera Krymskaya**

USA

**Piotr Kuna**

Poland

**Lisette Kunz**

Netherlands

**Thomasz Kuzniar**

USA

**Maria Teresa La Rovere**

Italy

**Ismail Laher**

Canada

**Lies Lahousse**

Belgium

**Katja Lakota**

Slovenia

**Lisa Langsetmo**

Canada

**Debra Laskin**

USA

**Maria Laucho-Contreras**

USA

**Heung-Man Lee**

South Korea

**Yong Chul Lee**

South Korea

**Pedro Leme**

Brazil

**Nanfang Li**

China

**Feng Li**

China

**Chunying Li**

USA

**Rebecca Lim**

Australia

**Rik Lories**

Belgium

**Jan Lotvall**

Sweden

**Renaud Louis**

Belgium

**Stelios Loukides**

Greece

**Jane Lucas**

UK

**Nicholas Lukacs**

USA

**Giuseppe Lungarella**

Italy

**Fayong Luo**

USA

**Xiongbiao Luo**

China

**Barbara Lutey**

USA

**Van Ly**

Australia

**Anjali Lyengar**

USA

**Roberto Machado**

USA

**Tania Maes**

Belgium

**Poornima Mahavadi**

Germany

**Arnaud Mailleux**

France

**Patrick Mallia**

UK

**Peter Mancuso**

USA

**Nilam Mangalmurti**

USA

**David Mannino**

USA

**Stefan Marciniak**

UK

**James Martin**

Canada

**Sadis Matalon**

USA

**Alexander G Mathioudakis**

Greece

**Hisako Matsumoto**

Japan

**Kazuto Matsunaga**

Japan

**Gustavo Matute-Bello**

USA

**Eric Maury**

France

**Armand Mekontso Dessap**

France

**Javier Mendez**

USA

**Paul Mercer**

UK

**Mervyn Merrilees**

New Zealand

**Tesfaye Mersha**

USA

**Herman Meurs**

Netherlands

**Stefan Mihaicuta**

Romania

**Hielko Miljoen**

Belgium

**Laura Millares**

Spain

**Roxana Mincheva**

Sweden

**Massimo Miniati**

Italy

**Marc Miravitlles**

Spain

**Makoto Miyara**

France

**Lyn Moir**

Australia

**Subhabrata Moitra**

USA

**Valentina Monica**

Italy

**Paolo Montuschi**

Italy

**Bethany Moore**

USA

**Thierry Moreau**

France

**Floriana Morgillo**

Italy

**Rory Morty**

Germany

**Joachim Mueller-Quernheim**

Germany

**Shigeo Muro**

Japan

**Steven Mutsaers**

Australia

**Jean-Marc Naccache**

France

**Robert Naeije**

Belgium

**Anjaparavanda P Naren**

USA

**Alfons Navarro**

Spain

**Mehdi Nikkhah**

USA

**Jake Nikota**

Canada

**Hilario Nunes**

France

**George Nutsios**

USA

**Shinya Ohkouchi**

Japan

**Olav Oldenburg**

Germany

**Brian Oliver**

Australia

**Dario Olivieri**

Italy

**Karen Olsson**

Germany

**Henric Olsson**

Sweden

**Christian Opitz**

Germany

**Victor Ortega**

USA

**Pierluigi Paggiaro**

Italy

**Nikos Papadopoulos**

UK

**Annie Pardo**

Mexico

**Andrew Paris**

USA

**Ian Pavord**

UK

**Gunnar Pejler**

Sweden

**Raymond Penn**

USA

**Tonio Pera**

USA

**Francisco Perez-Vizcaino**

Spain

**Charles Pilette**

Belgium

**Maria Pino-Yanes**

Spain

**Francesco Pistelli**

Italy

**Carole Planes**

France

**Laurent Plantier**

France

**Chiara Poggi**

Italy

**Francesca Polverino**

USA

**Mateo Porres-Aguilar**

USA

**Lars Poulsen**

Denmark

**Douglas Powell**

USA

**YS Prakash**

USA

**Naga Prasad**

USA

**Mirella Profita**

Italy

**David Proud**

Canada

**Sharen Provoost**

Germany

**Moyar Qing Ge**

USA

**Christophe Quesnel**

France

**Roberto Rabinovich**

UK

**Marko Radic**

USA

**Madeleine Rådinger**

Sweden

**Irfan Rahman**

USA

**Scott Randell**

USA

**Deepa Rastogi**

USA

**Marianne Ratcliffe**

UK

**Lara Ravanetti**

Netherlands

**Elisabetta Renzoni**

UK

**Sebastian Reuter**

Germany

**Niki Reynaert**

Netherlands

**Paul Reynolds**

USA

**Carla Ribeiro**

USA

**Fabio Ricciardolo**

Italy

**Barbara Rinaldi**

Italy

**Esra Roan**

USA

**Patricia Robbe**

Netherlands

**Mary Robbins**

USA

**Josep Roca**

Spain

**Nicolas Roche**

France

**Maria Roditis**

USA

**Gernot Rohde**

Netherlands

**William Rom**

USA

**Jesse Roman**

USA

**Freddy Romero**

USA

**Giulio Rossi**

Italy

**Antonio Rossi**

Italy

**Michael Roth**

Switzerland

**Alex Rothman**

UK

**Robbert Rottier**

Netherlands

**Frans Rutten**

Netherlands

**Erica Rutten**

Netherlands

**Anderson Ryan**

UK

**Kristina Rydell-Törmänen**

Sweden

**Yogesh Saini**

USA

**Umadevi Sajjan**

USA

**Yuji Sakuma**

Japan

**Daniel Salerno**

USA

**Jean Michel Sallenave**

France

**Konstantinos Samitas**

USA

**Venkatesh Sampath**

USA

**Raul Sansores**

Mexico

**Pierachille Santus**

Italy

**Ian Sayers**

UK

**Andrea Schamberger**

Germany

**Marie-Claire Schanne-Klein**

France

**Nicolas Schlegel**

Germany

**Mirco Schmolke**

Switzerland

**Richard Schulz**

Germany

**Nicola Scichilone**

Italy

**Jeremy Scott**

Canada

**Arti Shukla**

USA

**Don Sin**

Canada

**Cherie Singer**

USA

**Sally Singh**

UK

**Dave Singh**

UK

**Eleni Sofianopoulou**

UK

**Ronald Sorkness**

USA

**Arnold Spek**

Netherlands

**Jens Spiesshoefer**

USA

**Domenico Spina**

UK

**Luminita A Stanciu**

UK

**Hisatoshi Sugiura**

Japan

**Bela Suki**

USA

**Nicola Sverzellati**

Italy

**Tsutomu Tamada**

Japan

**Dale Tang**

USA

**Claudio Tantucci**

Italy

**Sadatomo Tasaka**

Japan

**Amanda Tatler**

UK

**Christian Taube**

Netherlands

**Luc Teppema**

Netherlands

**Luis Teran**

Mexico

**Gabriel Thabut**

France

**Brack Thomas**

Switzerland

**Neil Thomson**

UK

**Omar Tliba**

USA

**Masako To**

Japan

**Kjell Toren**

USA

**Antoni Torres**

Spain

**Nahev Tov**

Israel

**Bruce Trapnell**

USA

**Francois Trottein**

France

**Hoi Nam Tse**

Hong Kong

**Lena Uller**

Sweden

**Yurdagul Uzunhan**

France

**Isabelle Vachier**

France

**Job FM van Boven**

Netherlands

**Marianne van de Pol**

Netherlands

**Joost van den Aardweg**

Netherlands

**Sanne van Kampen**

Netherlands

**Chris van Weel**

Netherlands

**Jeroen Vanoirbeek**

Belgium

**Katerina Vaporidi**

Greece

**Alessandro Vatrella**

Italy

**Fien Verhamme**

Belgium

**Sandra Viera**

Brazil

**Sarah Vierkotten**

Denmark

**Nils von Neuhoff**

Germany

**Marie Catherine Vozenin**

Switzerland

**Shilpa Vyas-read**

USA

**Darcy Wagner**

Germany

**Lichun Wang**

USA

**Yuelan Wang**

China

**Brian Ward**

Canada

**Norbert Weissmann**

Denmark

**Zhenke Wen**

China

**Ching-Feng Weng**

Taiwan

**Chris Weston**

UK

**Kenneth Witwer**

USA

**Martin Witzenrath**

Germany

**Michael Wolin**

USA

**Cherry Wongtrakool**

USA

**Damo Xu**

UK

**Mitsuhiro Yamada**

Japan

**Masao Yamaguchi**

Japan

**Nadir Yehya**

USA

**Akihito Yokoyama**

Japan

**Jason Yuan**

USA

**Amir Zeki**

USA

**Ping Zhu**

China

**Torsten Zuberbier**

Germany

